# Effectiveness and Acceptability of a Sexual Health Education Program for Muslim Countries: An Intervention Study

**DOI:** 10.1007/s10508-025-03111-8

**Published:** 2025-03-17

**Authors:** Özlem Karatana, Abdullah Beyhan, Ayşe Ergün

**Affiliations:** 1 Nursing Department, Doğuş University, 34775 Istanbul, Turkey; 2 Nursing Department, Siirt University, Siirt, Turkey; 3 Nursing Department, Marmara University, Istanbul, Turkey

**Keywords:** Sexual health, Sexual behavior, Sexual health education program, Muslim university students

## Abstract

In Muslim societies, issues related to sexual and reproductive health are rarely discussed and considered sensitive subjects. This study was conducted to assess the acceptability and effectiveness of a sexual health education program in Turkey, a Muslim country. The study was conducted using a one-group quasi-experimental design. The sexual health education program consisted of five sessions of sexual health education, pre-test, post-test, first follow-up (30 days after) and second follow-up (60 days after). The sample of the study consisted of 207 female and 79 male first year Muslim university students. At the end of the program, 96.5% of the Muslim university students stated that they liked the education, 96.2% found the education useful, 86.0% found the online education convenient, and 96.9% would use the information they learned. Muslim university students, in general, found the feasibility of the education to be highly favorable. It was determined that the post-test score of sexual health knowledge was higher than the pre-test score (*p* < 0.05). The program in this study was found to be adopted by Muslim university students and it is thought to be valuable in societies with similar religious beliefs and views.

**Supplementary Information:**

The online version contains supplementary material available at 10.1007/s10508-025-03111-8.

## Introduction

Sustainable development goals and global health agendas recognize sexual health education as an important entry point for promoting adolescent health both as an end in itself and as a means of improving the overall health and well-being of young people. Cultural and religious factors play a crucial role influencing sexual health perceptions, sexual health knowledge and needs. For example, issues related to sexual and reproductive health are rarely discussed in Muslim societies and are considered sensitive subjects (Shirpak et al., 2008). Although Islam allows for the teaching or provision of safer sex information to individuals as a health promotion strategy, they do not appear to receive standardized education (Rosenfield, 1997; Shaw & El-Bassel, 2014). The world has long recognized the significance of sexuality education, and countries have made attempts at increasing public awareness by integrating it into their educational curricula. However, sexuality education remains an extremely controversial topic when policies and practices are largely dictated by traditional and cultural beliefs (Shaikh & Ochani, 2018). Therefore, Muslim young adults are compelled to resort to sources which might be of questionable authenticity, accuracy and appropriateness (Adamczyk & Hayes, 2012). However, their doubtful reliability makes young people susceptible to exploitation, harm and misinformation (Iqbal et al., 2017).

Islam has highly valued children's rights, particularly to education and health, and recognizes education regarding sexual matters as a crucial part of every child's upbringing. According to the Islamic faith, the ideal young Muslim is predominantly conceptualized as someone who undergoes an asexual transition from birth to puberty and remains sexually abstinent after puberty until marriage (Tabatabaie, 2015). Such a problematic position of childhood and adolescent sexuality in an Islamic perspective, as Tabatabaie (2015) notes, necessitates controlling and, more importantly, preventing childhood and adolescent sexualities in Muslim societies. For any educational intervention to work, it should be tailored to meet the target population’s specific needs. The United Nations Educational, Scientific and Cultural Organization’s International Technical Guidance on Sexuality Education emphasizes the need for sexual health education programs that are scientifically accurate, incremental in nature with a spiral curriculum approach, age-appropriate, and, most importantly, culturally and contextually relevant (UNESCO, 2018). There are two types of sexual health education adopted in the world: (1) sexual abstinence and (2) comprehensive sexual health education (Fields et al., 2015). The sexual abstinence education aiming to delay sexual intercourse until marriage is based on the lack of sexual experience of young individuals, and this education tends to be supported in contexts where religious beliefs are stronger and non-procreative sexual practices and partnerships are censured (Lemon, 2019; Miller, 2019). But it is important to note that the assumption that Muslim young adults are not sexually active is incorrect and leads to a failure to address their sexual health needs. Comprehensive sexual health education, on the other hand, explains to young individuals the benefits of delaying sexuality until they are emotionally and physically ready instead of giving the message of “avoiding sexual intercourse until marriage.” It also teaches young individuals how to protect themselves from infections and unwanted pregnancy when they decide to have sexual intercourse (AVERT, 2014). Therefore, it strengthens the argument that comprehensive sexual health education can assist Muslims young to abide by the religious ideal of premarital abstinence, rather than encouraging them to have premarital sex.

It is necessary to design sexual education programs that are religiously appropriate and accepted in Muslim societies. Muslim understandings of sexuality education remain largely obscure in both public and academic spheres because they are often ignored (Sanjakdar & Yip, 2017). Since sexual health is not taught in any formal setting in most Muslim countries, there is a need for new models of education, especially comprehensive sexual health education. This is because the lack of comprehensive sex education for Muslim young adults increases both the risks that they will have unsafe sex and the likelihood that they will make the decision to have premarital sex without being adequately informed of the possible consequences, and this information is essential to maintaining sexual health once they are married. A review of the literature reveals that sexual health education programs have been conducted in Muslim countries such as Iran, Ethiopia and Kenya (Fasil et al., 2022; Gaughran & Asgary, 2014; Mahmodi & Valiee, 2016). However, there appears to be no effective and acceptable sexual health education program for both male and female university students in Muslim countries. Accordingly, the aim of this study was to design a sexual health education program in a Muslim countries and to evaluate the acceptability and effectiveness of this program.

## Method

### Participants

In this study, 483 male and female students studying in the first year of a university in Turkey, a Muslim country, were invited. No sample selection was made, and it was aimed at reaching all first year students of the university. Since the study was voluntary, 197 students did not participate in the study because it was related to sexual health education, they did not have enough time, and other reasons. As a result, this study was completed with 286 female (*n* = 207) and male (*n* = 79) students.

Before the study began, its purpose was explained to the participants, and participants’ consent was obtained. Participants were also informed that they could exit the study at any time. Participants gave consent after being informed in the online questionnaire and voluntarily participated in the study and answered the questionnaire. No compensation was offered as an incentive to take the survey.

### Procedure

**Fig. 1:**
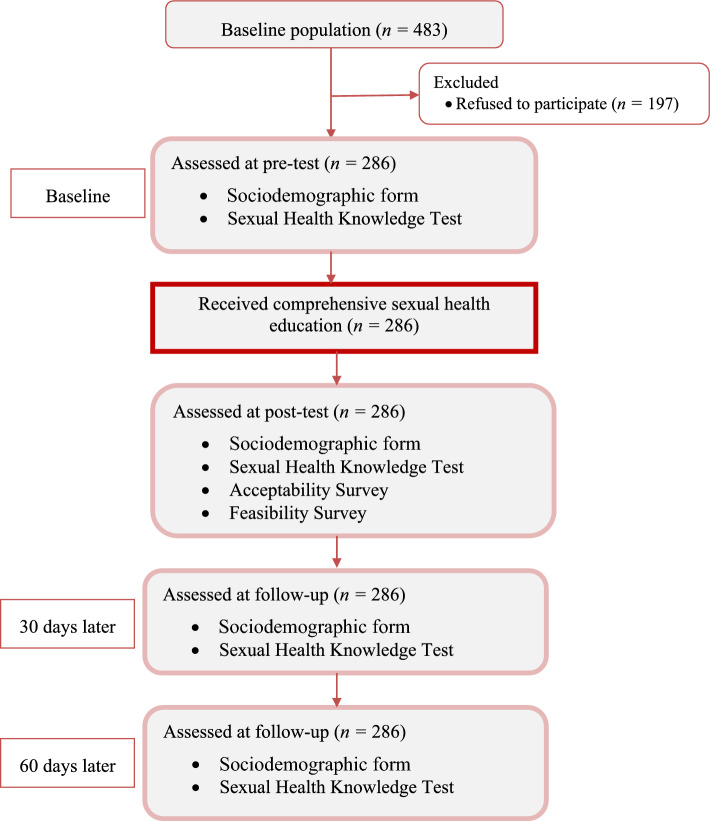
Process of study

This study was conducted between October and December 2020. The sexual health education program consisted of pre-test (pre-education), education initiative, post-test (post-education), first follow-up (30 days later) and second follow-up (60 days later). The flowchart of the study is given in Fig. 1. The sexual health education program was conducted in five online sessions on 5 consecutive days. The duration of each online session was different. The intervention content of the program is given in Table 1. The comprehensive sexual health education was provided in the sessions, and training topics were created in line with the literature (Latifnejad Roudsari et al., 2013; Sexuality Information and Education Council, 2009; Weaver et al., 2005). This program focuses on the right of Muslim young adults to receive comprehensive sex education that is religiously appropriate. The program was preferred to be an online program in terms of reaching a large number of Muslim young adults (Widman et al., 2020), protecting the sense of privacy, effective in reducing students' risky sexual behaviors (Kamke et al., 2020), easy access (Smith & Anderson, 2018) and low cost of education (Hall et al., 2016). Since all conversations about sexuality with Muslim university students must be within the context of Haya (sensitization of the heart and anguish when events that are contrary to decency occur) and to preserve this modesty, single-sex classes for sexuality education programs are preferred, as are classes taught by a teacher of the same sex (Ashraf, 1998), the education was given by the female educator to the female group and by the male educator to the male group via Zoom application. Zoom, a web-based application that provides quality audio, video and screen sharing, has created an interactive learning environment with features that enable them to record the education, watch them later and ask questions during the education.

**Table 1:** Content of the sexual health education program

Session topics	Aim	Intervention content	Number of participants
1. Session			
Physiology and anatomy of reproduction	Increasing the level of knowledge about female and male reproductive health	Message: How does sexual health affect overall health?	228 active
The importance of reproductive health	Understanding the effect of reproductive health on general health	30 min PowerPoint presentation (lecture, question–answer and discussion)	58 from records
2. Session			
Sexual development and lifelong sexuality	Increasing the level of knowledge about physical, sexual and psychosocial development stages	Message: Couples in romantic relationships can express their feelings to each other even without sexual intercourse (holding hands, hugging, expressing love in saying and explaining their feelings in writing)	238 active
Love/dating	Raising awareness about healthy relationships	45 min PowerPoint presentation (lecture, question–answer and discussion)	48 from records
Healthy romantic relationships			
3. Session			
Sexual behavior	Learning to live sexuality happily and safely, and gaining the necessary responsibilities	Message: Learning and adopting protective sexual behavior is important to maintaining sexual health	236 active
Protection methods		60 min PowerPoint presentation (lecture, question–answer and discussion)	50 from records
STD and AIDS/HIV			
4. Session			
Myths about sexuality	Recognizing false beliefs about sexuality and gaining correct knowledge	Message: Sexual life should be free from coercion and exploitation	230 Active
Sexual abuse and sexual violence		45 min PowerPoint presentation (lecture, question–answer and discussion)	56 from records
5. Session			
Decision-making skills	To be able to make the right decisions for a healthy sexual life and to learn personal hygiene methods in protecting sexual health	Message: Sexual decisions can affect an individual's future health and life plans	232 active
Personal hygiene and self-care		45 min PowerPoint presentation (lecture, question–answer and discussion)	54 from records

The sexual health education program was announced to the students through the social media groups of the university they were affiliated with. A WhatsApp group was created for students who gave consent to participate in this program. Before the session, short messages were sent via social media to increase participation. Computerized surveys were used before, after and during the follow-up.

### Measures

This study’s online survey consisted of three parts: (1) the sociodemographic form, (2) the Sexual Health Knowledge Test and (3) the Acceptability and Feasibility Survey. The data were collected via self-reports, and the survey took approximately 10 min to complete. The data were collected through an online questionnaire application.

#### Sociodemographic Form

This form consisted of 16 questions about individual characteristics (such as age, gender, marital status and sexual identity), opinion on premarital sexual experience, risky sexual behaviors (such as sexual behaviors, status of protection during sexual intercourse, status of STD testing and alcohol-induced sexual intercourse) and sexual abstinence (such as “are you sexually active? and have you ever had sexual intercourse that resulted in sexual intercourse?”).

#### Sexual Health Knowledge Test (SHKT)

It consisted of 40 multiple-choice questions covering sexual universal values, sexual identity development, sexual orientations, sex–gender, anatomy of reproductive system, sexual relationship/satisfaction, reproductive physiology, contraception, sexually transmitted infections, sexual violence and safe sexual behavior to determine students' knowledge about sexual and reproductive health (Evcili & Golbasi, 2017a, 2017b, 2019). For example, the universal values sub-dimensions: “Which of the following is not one of the universal values related to sexuality? a. Sexuality is unique to all human beings, b. Sexuality is a natural and healthy part of life, c. Every decision related to sexual life has effects and consequences, d. Sexual intercourse is the only way to express sexuality, e. Starting sexual behavior early brings risks.” The correct answer is d, etc. Each question has one correct answer. Each correctly responded item was given 1 point. Items not responded or responded incorrectly were given 0 points. The lowest and highest possible scores to be obtained from the test were 0 and 40, respectively. The higher the score, the higher the possibility of having sexual health knowledge. The widely used Item Discrimination Index reports the difference between the proportions of high and low scorers answering a dichotomous item correctly. High values are reputed to flag good items, low values bad. As a general rule, point-biserial values of 0.20 and above are considered to be desirable. As a result of the analyses, the final test consisting of 40 questions was obtained. Cronbach’s alpha coefficient of the 40 questions in the study was calculated as 0.88. The internal consistency of the scale was highly reliable. In this study, Cronbach's alpha was found to be 0.80.

#### Acceptability and Feasibility Survey

The program acceptability and feasibility questionnaire (Bauermeister et al., 2015; Widman et al., 2020) prepared in line with the literature was administered at the end of the education. The questionnaire consists of four questions evaluating the acceptability and nine questions evaluating the feasibility (four questions evaluating the content of the education and five questions evaluating the educators). It is a 5-point Likert type, ranging between strongly agree and strongly disagree.

### Statistical Analyses

The Statistical Package for the Social Sciences Version 26.0 (SPSS) was used to analyze the data obtained from this study. Normality of the distribution of the data was evaluated with the Kolmogorov–Smirnov test, which determined that the data were not normally distributed (Tabachnick & Fidell, 2021). Since the data did not demonstrate normal distribution, non-parametric tests were employed. Sociodemographic characteristics were evaluated using mean, standard deviation, frequency (n) and percentage distributions (%). The feasibility and acceptability of the program questionnaires were evaluated with frequency (n), percentage distributions (%) and chi-square. In the comparison of the pre-test, post-test, first follow-up and second follow-up values and differences of the questionnaires, the data were analyzed using the marginal homogeneity test, Wilcoxon signed-rank test with repeated measures and Friedman test were also used. The pre-test and post-test comparison for participants' sexual behavior was compared using the marginal homogeneity test for categorical data. The comparisons of average Sexual Health Knowledge and its sub-dimensions scores on the pre-test/post-test/follow-up were assessed with the Friedman test; the pre-test/post-test/follow-up test comparisons were assessed with the Friedman test. The Wilcoxon test was used in the evaluation of matched pairs. Using the bootstrapping (5.000 bootstrap samples) method, the confidence interval was determined to be 95%. Interpretation of the analysis resulted in an acceptable statistical significance level of *p* < 0.05.

## Results

The mean age of the participants was 20.04 ± 2.66 years, and 72.4% were females. It was determined that 21.0% of the participants (48.1% of males and 10.6% of females) had sexual intercourse, and the age of first sexual experience was 18.66 ± 2.56 (min = 14 and max = 24). About 58.4% of them stated that they did not receive sexual health education. Participants are described in Table 2.

**Table 2:** General and sexual health-related characteristics

Variables	Min.–Max	M ± SD
Age (*n* = 286)	17–24	20.04 ± 2.66
Age of first sexual experience (*n* = 286)	14–24	18.66 ± 2.56
Female (*n* = 207)	14–24	19.13 ± 2.31
Male (*n* = 79)	14–24	18.39 ± 2.68

	*n*	%
*Sex*		
Female	207	72.4
Male	79	27.6
*Marital status*		
Single	276	96.5
Married	10	3.5
*Gender identity*		
Heterosexual	260	90.9
Gay–Lesbian	2	0.7
Bisexual	2	0.7
Other	21	7.3
Not sure	1	0.3
*Having sexual health education*		
Yes	119	41.6
No	167	58.4
*Sexual experience*		
Yes	60	21.0
No	226	79.0
*First sexual experience* (*n* = 60)		
Primary–secondary school	2	3.3
High school	36	60.0
College	14	23.3
I am married–I did not have sexual intercourse before marriage	8	13.3
*Number of sexual partners in college* (*n* = 60)		
None	34	56.7
1	15	25.0
2–4	7	11.7
5–10	4	6.7
*Prevention methods* (*n* = 60)		
Long-acting method	2	3.3
Contraceptive pill	2	3.3
Condom	30	50.0
Morning-after pill	8	13.3
Withdrawal	18	30.0

Min: minimum, Max: maximum, M: mean and Sd: standard deviation

While the rate of sexual abstinence of the participants before the education was 63.3%, this rate was found to be 88.3% at the end of the education (*p* < 0.05). It was determined that the rate of having STD tests was 10% before the education and increased to 30% at the end of the education. The diagnosis increased from 1.7% to 11.7%, respectively (*p* < 0.05). While the rate of alcohol-induced sexual intercourse was 33.3% before the education, this rate decreased to 6.7% at the end of the education (*p* < 0.05). While 32.5% of the participants answered “I am completely against the premarital sexual experience of the woman” before the education, this rate increased to 53.5% at the end of the education. While 29.7% of the participants were completely against the premarital sexual experience of males before the education, this ratio increased to 49.7% at the end of the education (Table 3).

**Table 3:** Comparison of pre-test and post-test for sexual behaviors of the participants (*N* = 286)

Variables	Pre-testª		Post-test^b^		Test and *p* value	
	*n*	%	*n*	%	*χ*^2^	*p*
*Opinion on premarital sexual experience of a female*						
I’m strongly against it	93	32.5	153	53.5	6.63*	< .001
I perceive it as normal	130	45.5	114	39.9		
Undecided	63	22.0	19	6.6		
*Opinion on premarital sexual experience of a male*						
I’m strongly against it	85	29.7	142	49.7	6.41*	< .001
I perceive it as normal	140	49.0	123	43.0		
Undecided	61	21.3	21	7.3		
*Protection in sexual intercourse*						
Yes	38	63.3	38	88.3	3.08*	< .001
No	12	20.0	1	2.3		
Occasionally	10	16.7	4	9.4		
*STD testing status*						
Yes	6	10.0	18	30.0	− 2.55*	< .001
No	54	90.0	42	70.0		
*STD diagnosis*						
Yes	1	1.7	7	11.7	− 2.12*	< .01
No	59	98.3	53	88.3		
*Using alcohol and having sexual intercourse*						
Yes	20	33.3	4	6.7	4.00*	< .001
No	40	66.7	56	93.3		

*χ*^2^** = **Marginal homogeneity, ªPre-education and ^b^Post-education, *p < .05

A statistically significant difference was found between sexual health knowledge pre-test, post-test, first follow-up and second follow-up scores (χ^2^ = 124.07, *p* < 0.05). In the paired analysis, it was determined that the post-test score was higher than the pre-test (*Z* = -10.08, *p* < 0.05), and the first follow-up score was lower than the post-test (*Z* = -5.63, *p* < 0.05). There was no difference between the first follow-up and second follow-up scores (*Z* = -. 84, *p* > 0.05) (Table 4).

**Table 4:** Comparison of SHKT and sub-dimension pre-test, post-test, 1st follow-up, and second follow-up mean scores of the participants (*N* = 286)

Subscale of sexual health knowledge test (test min–max score)	SHKT					
	Pre-testª	Post-test^b^	1st follow-up^c^	2nd follow-up^d^	χ^2^/*p*	Paired comparison
	Mean ± SD	Mean ± SD	Mean ± SD	Mean ± SD		Wilcoxon test
Sexual universal values (0–2)	1.27 ± 0.78	1.57 ± 0.63	1.52 ± 0.64	1.50 ± 0.68	44.32*	b,c > a
Sexual identity development (0–4)	1.86 ± 0.96	2.54 ± 1.13	2.49 ± 1.11	2.39 ± 1.09	96.85*	b,c > a
Sexual orientations (0–3)	1.68 ± 0.89	1.91 ± 0.93	1.77 ± 0.97	1.73 ± 0.96	23.61*	b > a,c,d
Sex–gender (0–3)	2.11 ± 0.85	2.21 ± 0.84	2.09 ± 0.95	2.15 ± 0.90	8.53*	b > a,c
Anatomy of reproduction system (0–3)	1.50 ± 0.96	1.67 ± 0.93	1.58 ± 0.93	1.59 ± 0.94	7.78*	b > a
Sexual relationship satisfaction (0–4)	2.61 ± 1.01	3.08 ± 1.04	2.91 ± 1.09	2.90 ± 1.13	60.14*	b,c > a
Reproductive physiology (0–3)	1.35 ± 0.72	1.62 ± 0.86	1.50 ± 0.84	1.55 ± 0.82	33.80*	b,c > a
Contraception (0–6)	3.44 ± 1.36	4.04 ± 1.47	3.9 ± 1.51	3.91 ± 1.57	56.28*	b,c > a
Sexually transmitted infections (0–7)	3.46 ± 1.40	4.17 ± 1.64	3.90 ± 1.75	3.91 ± 1.62	56.61*	b,c > a
Sexual violence (0–3)	2.13 ± 0.88	2.23 ± 0.94	2.09 ± 1.02	2.09 ± 1.02	9.06*	b > c
Safe sexual behavior (0–2)	0.84 ± 0.68	1.04 ± 0.74	0.87 ± 0.73	0.81 ± 0.74	25.18*	b > a,c,d
SHKT total (0–40)	22.31 ± 5.70	26.13 ± 7.20	24.69 ± 7.78	24.58 ± 7.78	124.07*	b > a,c

*χ*^2^ = Friedman test, ªPre-education, ^b^Post-education, ^c^Post-education (30th day) and ^d^Post-education (60th day), *p < .05

Except for the sexual violence sub-dimension, the post-test scores of the sub-dimensions were significantly higher than the pre-test (*p* < 0.05). At the same time, the first follow-up score was significantly higher than the pre-test in sub-dimensions of universal values, development of sexual identity, sexual satisfaction, reproductive physiology, contraception and STD. A significant decrease was found in the sub-dimensions of sexual orientations, gender, sexual violence and safe sexual behavior during the follow-up periods (Table 4).

### Acceptability

In this study, 96.5% of the participants stated that they liked the education, 96.2% found the education useful, 86.0% found the online education convenient and 96.9% would use the information they learned. The acceptability of the program did not make a significant difference in terms of gender (Suppl. Table S1).

Participants, in general, found the feasibility of the education to be highly favorable. Male participants found the duration of education more adequate than female participants. No technical or user-related problems were encountered during data collection and education. Participants were highly satisfied with the presentation, tone of voice, speed of expression, use of understandable language and communication by the educators (Sup. Table S1).

## Discussion

The Islamic faith does not forbid sexual health education; on the contrary, this form of education is both acknowledged and expected (Quran, 2020). The important thing is to design a sexual health education program that is appropriate for Muslim societies. The aim of this study was to design a sexual health education program in Muslim countries and to evaluate its acceptability and effectiveness. According to our study results, it was determined that the sexual health education program was found to be acceptable and useful by Muslim university students, and had high feasibility in terms of education duration, tools, methods and content, increased the knowledge level of Muslim university students, it is considered to be an effective and feasible sexual health program to improve the sexual health of Muslim university students.

As observed in the previous research (Albanghali & Othman, 2020; Di̇şsi̇z et al., 2020; Farahani, 2020; Yanıkekrem & Üstgörül, 2019), in this study, it was determined that more than half of the Muslim university students had not received sexual health education before, and their sexual health knowledge level was moderate. In the systematic review conducted by Alomair et al. (2020), studies conducted with female university students in 13 Muslim countries were examined, and Muslim women had poor knowledge regarding STI signs and symptoms, prevention, diagnosis and treatment, in addition to many misconceptions. These results highlight the need for culturally sensitive sexual health education program for Muslim university students.

Our study supports the general finding in the previous research (Farahani, 2020; Fatemi et al., 2024; Nadeem et al., 2021) that a low percentage of people are sexually active before marriage. Premarital sex that occurs without young people being equipped with the knowledge and skills to safely negotiate sexual relationships also represents an imminent threat to their health. Although premarital sex is not accepted in Muslim societies and ignorance about sexuality and sexual risks may negatively affect Muslim individuals' safe sex practices during their first contact with sex. Consequently, there is a real need to inform and prepare Muslim university students for their first experiences of sexual intercourse. The abstinence-only model tends to be supported in contexts where religious beliefs are stronger and non-procreative sexual practices and partnerships are censured (Horanieh et al., 2020). However, in our study, considering that Muslim students may be sexually active, comprehensive sexual health education was provided, monogamy was emphasized, and the religious dimension of premarital intercourse was used. This training is thought to support the effectiveness of the program.

In this study, the Muslim university students stated that they liked the online sexual health program, found it appropriate and useful and would use the information they learned. In the study conducted by Bauermeister et al. (2015), it was stated that the participants found the programs that included online health education appropriate in terms of privacy and acceptability. In this study, the program was highly acceptable, and the positive results obtained during the follow-up period show that the Muslim university students used the information they learned. The comprehensive content of the program and the ability to access the program without the time and place limit with the ability to watch the record (at home, by mobile phone, computer, etc.) can be considered as factors that increase acceptability and satisfaction. In addition to the easy accessibility of online programs, the communication of the educator (Ghamdi et al., 2016), the comprehensibility of the language (Dessel et al., 2017) and competence (De Metz & Bezuidenhout, 2018) have an impact on the success of the program (Borawski et al., 2015). In this study, the Muslim university students stated that they were highly satisfied with the presentation, tone of voice, speed of expression, use of understandable language and communication of the educators. It is thought that choosing gender-specific educators is beneficial in increasing the effectiveness of the program by making the students feel more comfortable during education and asking their questions easily.

Studies confirm the effectiveness of religious beliefs and faiths on appropriate sex education and controlling and preventing sexual relationships before marriage or out of wedlock. In a review article, Bahrami et al. (2022) confirmed the significant role of religion in individuals’ beliefs, especially in discouraging adolescents from having sexual relationships before marriage. In the study, at the end of the sexual health education program, the protection rate of Muslim university students during sexual intercourse increased at the end of the program, while the rate of having alcohol-induced sexual intercourse decreased in our study. In addition, the increase in the rate of participants who had STD tests and were diagnosed after education was evaluated as the positive contribution of the program in terms of early diagnosis and treatment. The increase in the rate of Muslim university students who answered “I am completely against the sexual intercourse experience before marriage” after the program was found to be important in terms of adopting one sexual partner. Islamic law considers sexual intercourse outside legal marriage to be adultery and a sin (Khaerunisa, 2021).

After the program, all sub-dimension post-test scores of the Muslim university students except total sexual health and sexual violence were found to be higher than the pre-test. In addition, the first follow-up score was higher than the pre-test in sub-dimensions of universal values, development of sexual identity, sexual satisfaction, reproductive physiology, contraception and STD. However, relapses were observed during the follow-up periods in sub-dimensions of sexual orientations, gender, sexual violence and safe sexual behavior. These results show that additional methods are needed to ensure the long-term permanence of education. In the future studies, especially considering the relapsed sub-dimensions, it may be suggested to increase the permanence of education with additional applications such as updating programs or text messages.

### Strengths and Limitations

There were several limitations in this study. This study used only Muslim university students and that they were selected through an online sampling technique, so the question remains whether or not the results can be generalized to other populations. In studies on sexuality in Muslim countries, it is not known whether what people say about their sexuality corresponds to what they actually do. Social desirability may have affected participant responses, especially because there was no control group for the intervention. In addition, the responses to the scales are based on self-report. This may lead to response bias.

### Conclusions

According to our study results, it was determined that more than half of Muslim university students did not receive sexual health education before university education. Young Muslims should be educated on sexual health issues at an early age, and services should be provided to those in need. Therefore, there is a need for sexual health programs that are acceptable to both countries and young Muslims. The aim of this study was to design a sexual health education program in Muslim countries and to evaluate its acceptability and effectiveness. It showed that this sexual health education program was applied to Muslim students and achieved the desired results. As a result of this program, it is thought that the knowledge level of Muslim university students increased, and the participants found the program acceptable and useful within the framework of their religious beliefs and that it is an effective and applicable sexual health program to improve their sexual health.

The sexual health education program designed and implemented in this study will be of value in communities with similar religious beliefs and views. Further study in different settings is needed to evaluate the feasibility and effectiveness of the intervention for scale-up. In addition, the acceptability and effectiveness of this program can be investigated by applying it to Muslim high school students.

## Supplementary Information

Below is the link to the electronic supplementary material.

Supplementary file1 (DOCX 16 kb)
