# Supplementary material for: Effectiveness and Acceptability of a Sexual Health Education Program for Muslim Countries: An Intervention Study
Source: Arch Sex Behav. 2025 Mar 17;54(4):1617–26. doi: 10.1007/s10508-025-03111-8 (PMC12011900; doi:10.1007/s10508-025-03111-8)
Supplement: Supplementary file 1 — Supplementary file1 (DOCX 16 kb) [file 10508_2025_3111_MOESM1_ESM.docx]

**Sup Table 1** Participants' view on the acceptability and feasibility of the program

|  | **Female**  **n=207** | **Male**  **n=79** | **Between-Group Comparison** | |
| --- | --- | --- | --- | --- |
| **Acceptability** | **% (n)** | **% (n)** | **χ^2^** | ***p*** |
| Liked Program | 97.6 (202) | 93.7 (74) | 2.59 | 0.14 |
| Learned New Things | 97.6 (202) | 92.4 (73) | 4.14 | 0.07 |
| Suitable for online education | 87.4 (181) | 82.3 (65) | 1.26 | 0.35 |
| Will use information | 98.1 (203) | 93.7 (74) | 3.62 | 0.12 |
| **Feasibility** |  |  |  |  |
| **Evaluation of Education** |  |  |  |  |
| The duration of the training was sufficient | 80.2 (166) | 92.4 (73) | 6.20 | 0.02 |
| Tools used in education was sufficient | 93.7 (194) | 92.4 (73) | 0.15 | 0.89 |
| Training methods were appropriate | 96.6 (200) | 93.7 (74) | 1.23 | 0.32 |
| It was sufficient to cover the topics in the training program. | 91.8 (190) | 93.7 (74) | 0.28 | 0.77 |
| **Educator's Assessment** |  |  |  |  |
| The presentation of the educator was good | 97.6 (202) | 93.7 (74) | 2.59 | 0.14 |
| The tone of the educator was sufficient | 98.6 (204) | 93.7 (74) | 5.00 | 0.03 |
| The instructor's narrative speed was appropriate | 97.6 (202) | 92.4 (73) | 4.14 | 0.07 |
| The language used by the educator was understandable | 97.6 (202) | 93.7 (74) | 2.59 | 0.14 |
| The communication of the educator was appropriate | 97.1 (201) | 93.7 (74) | 1.80 | 0.18 |

**Note.** For each group, the % (n) refers to the number of students who reported “4” or “5” to that item.
